# Metformin as a Potential Neuroprotective Agent in Prodromal Parkinson's Disease—Viewpoint

**DOI:** 10.3389/fneur.2020.00556

**Published:** 2020-06-12

**Authors:** Carolina Sportelli, Daniele Urso, Peter Jenner, K. Ray Chaudhuri

**Affiliations:** ^1^National Parkinson Foundation International Centre of Excellence, King's College Hospital, London, United Kingdom; ^2^Institute of Psychiatry, Psychology & Neuroscience, King's College, London, United Kingdom; ^3^Institute of Pharmaceutical Sciences, Faculty of Life Sciences and Medicine, King's College, London, United Kingdom

**Keywords:** Parkinson's disease, prodromal, metformin, neuroprotection, idiopathic REM behavior disorder

## Abstract

To date, there are no clinically effective neuroprotective or disease-modifying treatments that can halt Parkinson's disease (PD) progression. The current clinical approach focuses on symptomatic management. This failure may relate to the complex neurobiology underpinning the development of PD and the absence of true translational animal models. In addition, clinical diagnosis of PD relies on presentation of motor symptoms which occur when the neuropathology is already established. These multiple factors could contribute to the unsuccessful development of neuroprotective treatments for PD. Prodromal symptoms develop years prior to formal diagnosis and may provide an excellent tool for early diagnosis and better trial design. Patients with idiopathic rapid eye movement behavior disorder (iRBD) have the highest risk of developing PD and could represent an excellent group to include in neuroprotective trials for PD. In addition, repurposing drugs with excellent safety profiles is an appealing strategy to accelerate drug discovery. The anti-diabetic drug metformin has been shown to target diverse cellular pathways implicated in PD progression. Multiple studies have, additionally, observed the benefits of metformin to counteract other age-related diseases. The purpose of this viewpoint is to discuss metformin's neuroprotective potential by outlining relevant mechanisms of action and the selection of iRBD patients for future clinical trials in PD.

## Introduction

PD is the fastest growing neurodegenerative age-related disorder with numbers of patients projected to double by 2040 globally (1). The neuropathology is complex and mainly characterized by two features, a selective degeneration of dopaminergic neurons in the *substantia nigra pars compacta* (SNpc) and the presence of fibrillar aggregates referred to as Lewy bodies (LBs), mainly composed of α-synuclein, which manifest in motor and non-motor features (2–4). According to Braak's hypothesis, the progressive accumulation of α-synuclein-rich LBs begins in the medulla oblongata and anterior olfactory structures and progresses in a stereotypical bottom-up caudo-rostral direction to the neocortex (5). This concept is concordant with the now recognized prodromal state of PD, in which the ongoing pathological process confined to the lower brainstem areas are associated only with some specific non-motor/pre-motor features of PD (6). Many would therefore argue that the emergence of these symptoms along with some genetic markers in susceptible individuals could mark the beginning of PD.

Despite a massive effort involving preclinical investigations and post-mortem studies, the precise pathogenic mechanisms remains unclear and no unifying mechanism has been discovered to account for neurodegeneration in PD (7). It is likely that PD may occur as a result of multiple variable processes involving a range of pathogenic mechanisms. These include, mitochondrial dysfunction, oxidative stress, protein aggregation, abnormal protein degradation due to alterations in proteostasis mechanisms, neuroinflammation, and aging (8–10). While many of these processes might initiate the pathogenic process, by the time motor symptoms become manifest, a cascade of biochemical events leading to cell death will have become engaged. This might explain why clinical trials aimed at early intervention for neuroprotection or neuromodulation, based on preventing discrete components of this cascade, have consistently failed in the face of the already established widespread pathological change and a maelstrom of disruption of cellular functions. At present there are no clinically effective neuroprotective or disease-modifying strategies available for PD and this remains one of the defining unmet needs in the management of this challenging disorder (11, 12). Worryingly, a plethora of potential neuroprotective agents have been produced on the back of *in vitro* models of dopaminergic cell death and positive effects in *in vivo* animal models of the presumed pathogenic processes occurring in PD, but none have translated into effective treatments in man (13, 14). Over a billion US dollars have been spent by charity and industry to fund, develop and validate neuroprotective treatment strategies but to no avail.

## The Failure of Current Clinical Trial Designs

An alternative explanation for the failure to develop neuroprotective or disease-modifying strategies may rest with the design of clinical studies as these presume that all patients with PD have identical pathogenic mechanisms underlying their disease, which is unlikely to be true. In addition, most neuroprotective trials in PD have enrolled patients either in a “*de novo* untreated stage” or “early stable treated stage” in attempts to intervene at a point where the rescue of neurons is still feasible—yet the results have been uniformly negative (15). This approach may be flawed as post-mortem studies show that by 4 years after a clinical diagnosis of PD, there is already virtually complete nigrostriatal denervation of the dorsal putamen, profound nigral cell loss and abundant Lewy pathology, which raises the question of the possibility to achieve neuroprotection even in these “early motor stages” (16). It is thus crucial to understand when to intervene, taking into account the natural history pattern of PD, which develops from a pre-prodromal and prodromal stage progressing through stable and unstable phases to a palliative stage (17) (Supplementary Figure 1).

A body of clinical and pathological evidence support the concept of a prodromal stage of PD existing several years, maybe decades, prior to formal PD diagnosis (6, 18). This prodromal stage theoretically represents the ideal time point during which neurodegeneration has just commenced and restoration or protection is still feasible (19). Indeed, recent observations suggest that mitochondrial dysfunction, increased glycolysis and neuroinflammation occur in the prodromal stage of PD (20). In addition, most PD studies continue to mainly focus on motor endpoints despite PD being recognized to also be a non-motor disorder, with a complex range of non-motor symptoms (NMS) that span from prodromal to advance stages of the disease (21). These include dysphagia, autonomic dysfunction, sleep disorders, mood disturbances, cognitive impairment, and dementia (22, 23). This raises the possibility that undertaking clinical trials in the prodromal phase could be an essential step for investigating disease progression and testing agents with putative neuroprotective or disease modifying effects. In addition, drug repurposing could represent an interesting source of candidates to treat or slow diseases as costs are considerably lower than those needed for designing and optimizing a new drug. Furthermore, the safety and tolerability of many repurposed molecules is likely to have been already been established, making clinical trials more cost-effective and in need of smaller samples sizes. To date, drug repurposing have shown many advantages mostly in symptom management of disease progression (24). A drug has yet to be found that can fully revert or prevent the mechanisms of neurodegeneration, however anti-diabetic drugs have been proven to be safe and potentially effective in the treatment of PD.

## Association Between Type 2 Diabetes Mellitus and PD

An association between type 2 diabetes mellitus (T2DM) and PD has previously been reported (25), although meta-analyses of prospective cohort studies now suggest that the risk of developing PD among diabetic patients is quite low (26). Patients with both T2DM and PD present aggravated motor symptoms, higher degree of cognitive impairment and earlier onset of motor complications compared to non-diabetic subjects with PD (27–31). In addition, common pathogenic mechanisms also exist between PD and T2DM (32) and are listed in Table 1.

**Table 1:** List of common pathogenic mechanisms that may exist between PD and T2DM.

**PD link target**	**Possible mechanism**	**References**
Striatum	Impaired DA function in the dorsal striatum and impaired SN iron homeostasis	(33)
Peroxisome and mitochondria	Reduced expression of PGC-1α	(34)
Inflammation	Increased neuroinflammation	(35)
Amyloid and α-synuclein	Acceleration of α-synuclein amyloid fibril formation	(36)
*SNCA* gene in *SNCA*-deficient mice	Association between *SNCA* and insulin resistance	(37)
*DJ1* and *PINK1* genes	Dysfunction linked to insulin resistance in mouse models	(38) (39)

*From (32)*.

### Anti-diabetic Drugs and PD Progression

The potential effect of anti-diabetic drugs on the progression of PD has been assessed by several groups. For example, the use of thiazolidinediones was associated with a decreased risk of developing PD in diabetic patients (40) and a reduction of neurodegeneration and neuroinflammation in animal models (41–44). Incretin mimetic agents such as exenatide may also confer some degree of neuroprotection in functional models of PD (45–48). Exenatide has been shown to reduce dopaminergic cell death, improve motor and cognitive functions, decrease neuroinflammation and mitochondrial dysfunction (45, 49–51). A single-center randomized, double-blind, placebo-controlled trial showed that patients with moderate PD treated with exenatide once a week for 48 weeks had a significant 3.5-point advantage compared with those given placebo in the Movement Disorders Society Unified Parkinson's Disease Rating Scale (52). This study also observed some improvement in cognitive decline associated with PD (53, 54). Undoubtedly these initial findings are encouraging and also provide evidence that anti-diabetic agents may have a therapeutic or potentially a neuroprotective role in the treatment of PD. Furthermore, a national multicentre study addressing the potential of exenatide and neuroprotection has now begun in the UK to explore if the findings of the single-center study can be replicated. We posit that another well-established, well-tolerated anti-diabetic drug, metformin, with a long-established safety record should be also investigated in PD. Preclinical studies have shown that metformin may target most pathological mechanisms involved in PD and improve some aging outcomes. In addition to having a pleiotropic action, metformin has the advantage of being orally administered and thus preferential for patients to use over a long period of time, compared to subcutaneous injections needed to administer exenatide.

## Rationale for Selecting Metformin

Metformin is a cheap yet highly effective drug which has been used for over 50 years for the management of T2DM (55, 56). It is a synthetic dimethyl biguanide, orally administrated, which has been shown to reduce total mortality compared to other diabetes agents (57, 58). Metformin has a global safety record, is well-tolerated by the majority of patients and is used by roughly 125 million people worldwide (59). Metformin does not undergo significant metabolism and is excreted unchanged via the organic cation transporter-2 in the kidney (59, 60). Metformin does not have significant adverse effects and it has low risk for hypoglycaemia, however, it may cause vitamin B-12 deficiency (61) and lactic acidosis, mainly in patients with significant renal function impairment (62, 63). It is of important note that reported incidence of lactic acidosis in patients receiving metformin is very low and in over 20,000 patients exposed to metformin in clinical trials, there were no reports of lactic acidosis (64); diarrhea, nausea and stomach upset represent more common side effects (65).

**Figure 1:**
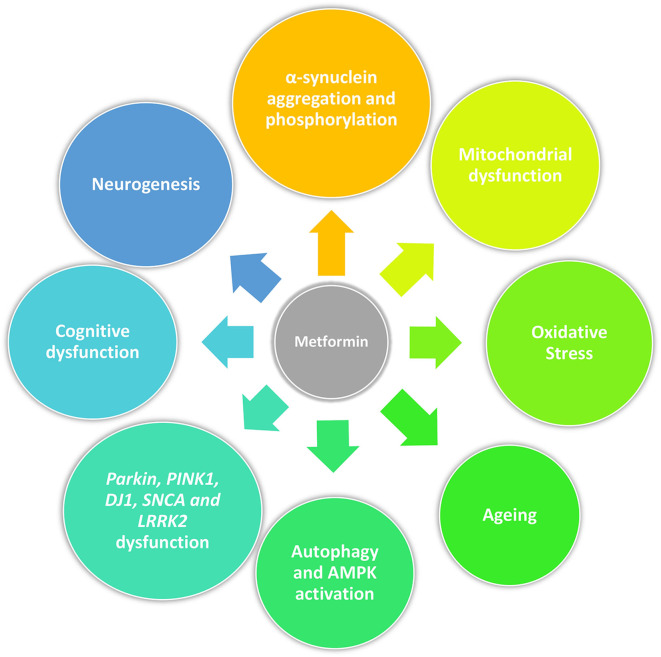
Pleiotropic action of metformin in PD. Beyond its anti-diabetic properties, metformin may act as a neuroprotective drug by reducing α-synuclein phosphorylation and aggregation, mitigating mitochondrial dysfunction and oxidative stress, influencing cellular processes associated with age-related conditions including cellular senescence and autophagy, and promoting neurogenesis. Furthermore, it could restore physiological molecular functions disrupted by genetic mutations related with PD (*Parkin, PINK1, DJ1, SNCA*, and *LRRK2*) and have an effect on cognition.

Beyond its anti-diabetic properties, metformin has a pleiotropic action and potentially slows aging by targeting mitochondrial metabolism and insulin signaling (66). Recent studies have demonstrated that metformin can rapidly penetrate the blood–brain barrier (67) and confer neuroprotection against stroke, cognitive impairment, Huntington's disease and potentially prevent dementia (68–73). Metformin can reduce α-synuclein phosphorylation and aggregation, influence cellular processes associated with age-related conditions including inflammation and autophagy, all of which are associated with PD pathogenesis. These actions are described in detail below as they may represent the potential of metformin to be neuroprotective or disease modifying in PD (74) (Figure 1).

## Mechanisms of Action of Metformin

### Mitochondrial Dysfunction

Mitochondrial dysfunction is commonly accepted as a key component of the pathogenesis of PD—through the inhibition of complex I and oxidative stress in sporadic disease and the linkage to *SNCA, parkin, PINK1, DJ-1*, and *LRRK2* mediated genetic forms of the illness (9, 75–77). Metformin also acts on mitochondria to alter the activity of the respiratory chain and to decrease reactive oxygen species (ROS) (78–81). This may have functional significance as metformin can protect dopaminergic cells against MPP^+^ toxicity *in vitro* by attenuating mitochondrial dysfunction and oxidative stress (82). Whether this translates *in vivo* is unclear as metformin has not consistently protected dopaminergic cells against toxin induced damage although it may reduce markers of oxidative stress—such as superoxide dismutase (83) and alter the expression of key mitochondrial proteins in basal ganglia (84). Metformin also restores the mitochondrial integrity of dopaminergic neurons disrupted by p*arkin* or *LRRK2* mutations in fruit flies (85). Importantly, metformin promotes the expression of peroxisome proliferator-activated receptor gamma coactivator 1α (PGC-1α) (84), a key regulator of mitochondrial biogenesis (86). PGC-1α is downregulated in the brain in PD and it protects dopaminergic neurones in animal models of PD (87). Although not directly focused on PD, metformin can alter mitochondrial fission and fusion protein expression and mitochondrial fragmentation in experimental systems linked to diabetes-induced oxidative stress, endothelial dysfunction, atherosclerosis development and Down's syndrome (88, 89). All of this being supportive of a potential neuroprotective role.

It is of important note that most of metformin's effects are an indirect result of complex I inhibition, although its exact mechanism warrants further investigation. Complex I alterations are widely associated with mitochondrial dysfunction and PD risk, as previously described in MPTP and rotenone models (90, 91). Despite this contradiction, however, sub-lethal concentrations of complex I inhibitors, such as metformin, which do not generate ROS (or produce a reduced amount), could still be beneficial and have a neuroprotective action (74).

### α-Synuclein Aggregation and Phosphorylation

The relationship between α-synuclein accumulation in LBs and neuronal toxicity is strong (92, 93). The evidence from familial *SNCA* mutations and PD in man coupled to the clear toxicity of fibrils and protofibrils of α-synuclein presents an opportunity for interfering in the final pathogenic process. Based on a range of experimental models of PD, metformin acts to counter the toxicity of the protein. In MPTP-treated mice, metformin reduced α-synuclein expression and the number of α-synuclein positive cells (82). In *C. elegans*, metformin reduced the loss of dopaminergic neurons and decreased α-synuclein aggregation induced by 6-hydroxydopamine (94). Recently, metformin treatment was shown to attenuate dopaminergic cell loss and α-synuclein accumulation in the SN of rotenone-treated mice (95). How metformin alters α-synuclein toxicity is not clear but the drug is able reduce the phosphorylation of the protein that is key to the mediation of its toxicity (96). This may relate to the ability of metformin to increase the activity of phosphatases involved in α-synuclein dephosphorylation as shown in the SN of MPTP-treated mice (97).

### Autophagy

Autophagy is a key cellular mechanism in protein homeostasis on which many of the pathogenic pathways involved in PD eventually converge (98). Autophagy plays a role in α-synuclein handling, mitochondrial function and oxidative stress, emphasizing its potential as a target for neuroprotection and disease modification (99, 100). Metformin has actions in experimental models of PD related to alterations in autophagy. For example, in MPTP-treated mice, metformin treatment prevented dopaminergic cell death and reduced motor impairment while decreasing α-synuclein aggregation, autophagic impairment, and ROS (82). The possible mechanism of this effect is not clear but may involve activation of AMP-activated protein kinase (AMPK) through the mitochondrial effects of metformin which in turn leads to an induction of autophagy involving in part, autophagosome formation and lysosomal biogenesis (101–104). Support for a role of AMPK comes from a study in MPTP-treated mice where downstream effectors of AMPK prevented dopaminergic cell death and motor impairment (105). This is supported by data from other areas, for example ischemia, where metformin exerts neuroprotective actions through manipulation of autophagy (106–108).

### Neurogenesis

Dopamine modulates ontogenetic neurogenesis (109). Post-mortem studies suggest that dopamine depletion may impair neuronal precursor cell proliferation in PD (109, 110) and thus negatively impact neurogenesis. While the relationships are not fully understood, impaired neurogenesis in the subgranular zone of the hippocampus and olfactory bulb of PD patients (109) likely contributes to memory deficits (111), depression (112), and olfactory dysfunction (113), commonly present in PD. Wang et al. (114) showed that metformin treatment could stimulate neurogenesis via an atypical PKC-CBP (Protein kinase C-CREB-binding protein) pathway. In particular, the transcription factor CREB (c-AMP response element-binding protein), was found to be a key component for neurodevelopment, cell survival, plasticity, memory and learning (115). Additionally, CREB was shown to regulate TH gene expression (116). At the molecular level, metformin may also upregulate the expression of the brain-derived neurotrophic factor (BDNF) by activating AMPK/CREB-mediated histone acetylation improving the ability of mice to resist stress (117). Therefore, activation of CREB by metformin could boost compensatory and regenerative mechanisms in the brain.

## Studies on Metformin Targeting Age-Related Diseases

Beyond the positive effects on multiple PD underlying mechanisms, metformin has also been shown to delay aging and extend lifespan in nematodes and rodents models (118–121). Metformin has, additionally, been considered to be effective in multiple human studies targeting age-related diseases. It was shown to delay cardiovascular disease, providing the rationale for metformin's designation as first-line therapy for most patients with T2DM UK Prospective Diabetes Study (UKPDS) Group (122). A recent study has shown lower mortality in patients with T2DM on metformin compared with non-diabetics despite the fact that the diabetic patients were more obese and had greater co-morbidities at baseline (123). Preliminary data support the concept that metformin may reduce the risk of cognitive impairment and dementia in both T2DM and non-T2DM (69, 72, 124). Long-term metformin therapy was also associated with lower incidence of neurodegenerative disorders among elderly veterans with T2DM (125). An ongoing trial called “Targeting Aging with Metformin” is validating metformin's ability to delay the onset of comorbidities related to aging (126). In relation to PD, clinical studies have mainly investigated the effects of metformin in comparison to, or in combination with other anti-hyperglycaemic agents and taken together all the studies look at different medications and are hardly comparable (40, 127). There is thus a lack of studies specifically evaluating the neuroprotective effects of metformin on PD development and/or progression.

## The Selection of an “Enriched” Cohort of Prodromal PD for a Metformin Trial

To confer neuroprotection against PD, in addition to understanding the cellular mechanisms involved in PD pathogenesis, it is crucial to perform studies in a group of patients that reside in the prodromal stage of the disease and who will most certainly phenoconvert to clinical PD within a reasonable time. The identification of such group would allow the testing of a putative neuroprotective or disease-modifying agent, such as metformin, in the ideal time frame to maximize the possible beneficial impacts (18).

The definition of this stage requires working with the probability of conversion to overt PD in large populations of “at-risk” subjects. These include patients with idiopathic rapid eye movement behavior disorder (iRBD), olfactory dysfunction, autonomic dysfunction, depression, excessive daytime sleepiness, constipation, or carriers of a known PD mutation such as LRRK2 or GBA. iRBD is defined as apparent acting out of dreams during REM sleep, associated with a loss of normal REM sleep atonia (128). iRBD has a strong evidence on being a predictor of synucleinopathies as multiple single-center prospective cohort studies have documented that iRBD phenoconverts to PD, dementia with Lewy bodies or multiple system atrophy in 80% of cases over a 6–10 years and possibly to a higher rate over 12 years (129–133). This risk is higher if a person with iRBD also displays other prodromal features of PD (olfactory dysfunction or constipation), shows a reduced uptake on presynaptic dopamine transporters (DaTSCAN) (134–137) or has mild bradykinesia. It is impossible to give a definitive conversion rate in iRBD patients as data are only available from a few highly selected cohort studies (136, 138). However, a likelihood ratio (LR) of several motor and non-motor features of phenoconversion to clinical PD is available (132, 139, 140). The LR is the highest for RBD, followed by a positive DaTSCAN and hyposmia (140). Thus, “enriched” iRBD cases (with hyposmia, or positive DaTSCAN) may provide an earlier window of phenoconversion and an optimal group to study neuroprotection (141). In addition, if such patients have mild bradykinesia, the time of phenoconversion is likely to be within 4 years as shown by a controlled study by Schrag et al. (142). We therefore would propose a randomized double blind placebo-controlled trial with metformin in an enriched iRBD cohort where other risk factors are also comorbid (hyposmia, abnormal DaTSCAN and/or bradykinesia) with a follow up period of 4–6 years to allow for the maximal possibility of phenoconversion to PD.

## Conclusion

To date, all clinical disease-modifying trials in PD have been performed in individuals in the manifested “in-life” motor stage, targeting either early “*de novo*” PD or treated PD and have all failed to show any convincing effects on neuroprotection. Investigating the prodromal phase of the disease could offer greater promise of success, assuming the less-advanced pathology and the greater potential to intervene at key points of the molecular pathogenesis. There are currently no studies using metformin to evaluate a possible protective effect on motor and non-motor functions on PD, although the idea seems compelling. Given its properties and the fact that mitochondrial dysfunction, autophagy, α-synuclein, aging, have all been proposed to be involved in PD pathophysiological processes, and the potential benefits of metformin to counteract age-related disorders (cancer, cardiovascular and neurodegenerative diseases), metformin seems a reasonable pluripotent agent to try given its safety, ease of use and wide availability. The above evidence encourages us to pilot a study investigating the role of metformin as a potentially neuroprotective agent in prodromal PD. iRBD subjects have a high likelihood to convert to PD or a related synucleinopathy and may therefore represent an ideal group for neuroprotective trials, enabling the field to push into investigating the prodromal stage of the disease and hopefully prevent or slow the development of PD.

## Data Availability Statement

All datasets generated for this study are included in the article/ Supplementary Material.

## Author Contributions

CS conceptualized the paper, drafted, and revised the manuscript. DU drafted and revised the manuscript. PJ revised the manuscript. KC conceptualized the paper and revised the manuscript.

## Conflict of Interest

The authors declare that the research was conducted in the absence of any commercial or financial relationships that could be construed as a potential conflict of interest.
