# Supplementary material for: Metformin as a Potential Neuroprotective Agent in Prodromal Parkinson's Disease—Viewpoint
Source: Front Neurol. 2020 Jun 12;11:556. doi: 10.3389/fneur.2020.00556 (PMC7304367; doi:10.3389/fneur.2020.00556)
Supplement: Supplementary file 1 [file Data_Sheet_1.DOCX]

**Supplementary Figure 1**. A schematic representation of the potential timepoints to start a neuroprotective trial and the corresponding populations, targets and endpoints to include. The graphic depicts the decline in dopaminergic neuronal function, the rates of development and progression of the motor (as reflected by the progression of Hoehn & Yahr stages) and non-motor burden, through the progressive stages of PD. The existence of a pre-prodromal stage can be hypothesised. Prodromal stage potentially starts when NMS appear. It is estimated that motor features emerge when approximately 50–60% of dopaminergic neurons have been lost (Gibb and Lees, 1991). At 4 years post-diagnosis, there is virtually complete dopaminergic denervation in the dorsal putamen (Kordower et al., 2013) (Note that the neuronal loss in PD is unlikely to follow a linear pattern). Adapted from (Titova and Ray Chaudhuri, 2018).

Gibb, W.R., and Lees, A.J. (1991). Anatomy, pigmentation, ventral and dorsal subpopulations of the substantia nigra, and differential cell death in Parkinson's disease. *Journal of neurology, neurosurgery, and psychiatry* 54**,** 388-396. doi: 10.1136/jnnp.54.5.388.

Kordower, J.H., Olanow, C.W., Dodiya, H.B., Chu, Y., Beach, T.G., Adler, C.H., et al. (2013). Disease duration and the integrity of the nigrostriatal system in Parkinson's disease. *Brain* 136**,** 2419-2431. doi: 10.1093/brain/awt192.

Titova, N., and Ray Chaudhuri, K. (2018). Non-motor Parkinson disease: new concepts and personalised management. *The Medical Journal of Australia* 208**,** 404-409. doi: 10.5694/mja17.00993.
