# Supplementary figures and images for: Metformin as a Potential Neuroprotective Agent in Prodromal Parkinson's Disease—Viewpoint
Source: Front Neurol. 2020 Jun 12;11:556. doi: 10.3389/fneur.2020.00556 (PMC7304367; doi:10.3389/fneur.2020.00556)

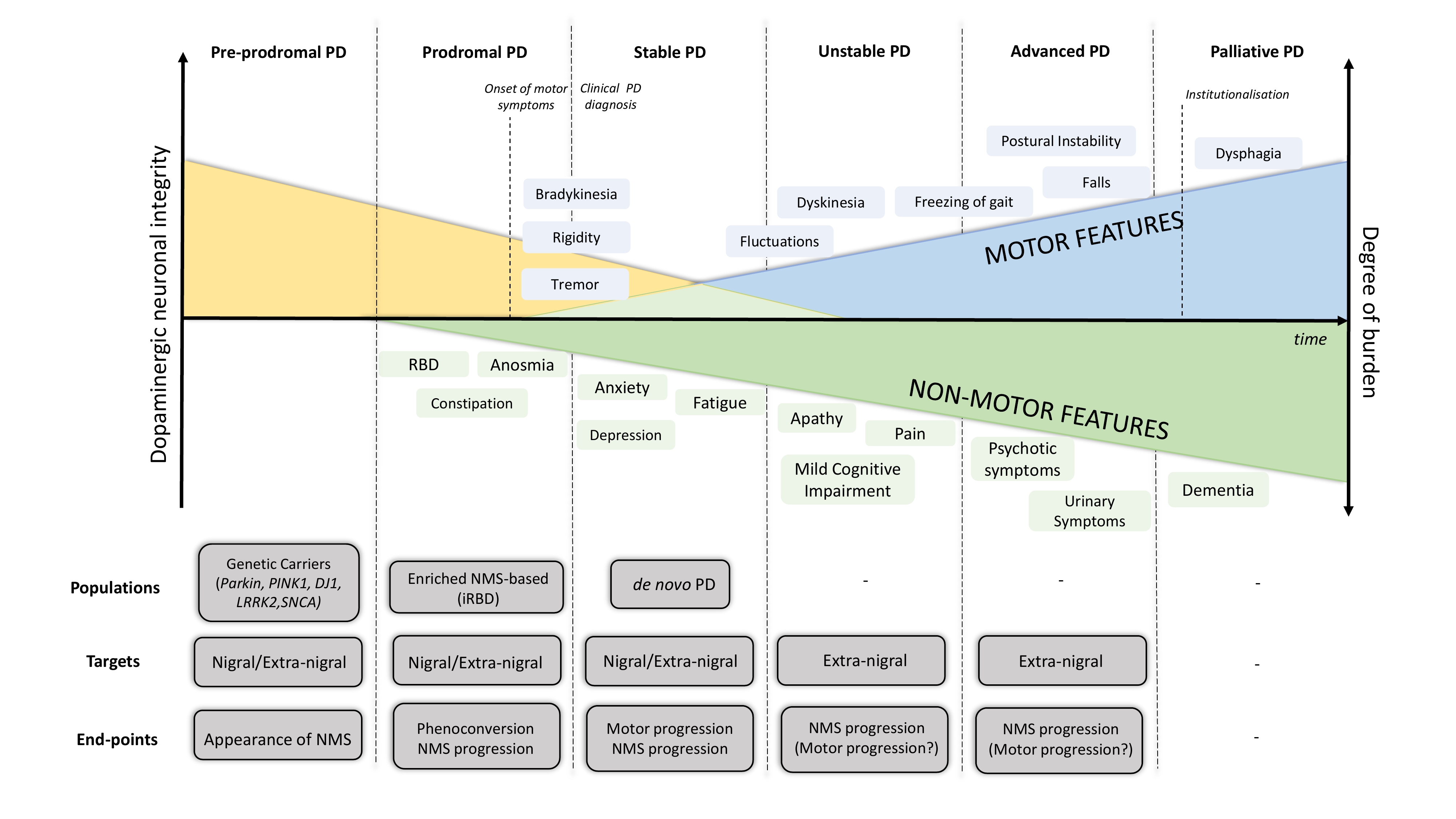

Supplement: Supplementary file 2 [file Image_1.JPEG]
